# Reagent and laboratory contamination can critically impact sequence-based microbiome analyses

**DOI:** 10.1186/s12915-014-0087-z

**Published:** 2014-11-12

**Authors:** Susannah J Salter, Michael J Cox, Elena M Turek, Szymon T Calus, William O Cookson, Miriam F Moffatt, Paul Turner, Julian Parkhill, Nicholas J Loman, Alan W Walker

**Affiliations:** Pathogen Genomics Group, Wellcome Trust Sanger Institute, Hinxton, UK; Molecular Genetics and Genomics, National Heart and Lung Institute, Imperial College London, London, UK; Institute of Microbiology and Infection, University of Birmingham, Birmingham, UK; Shoklo Malaria Research Unit, Mahidol-Oxford Tropical Medicine Research Unit, Faculty of Tropical Medicine, Mahidol University, Mae Sot, Thailand; Centre for Tropical Medicine, Nuffield Department of Medicine, University of Oxford, Oxford, UK; Microbiology Group, Rowett Institute of Nutrition and Health, University of Aberdeen, Aberdeen, UK

**Keywords:** Contamination, Microbiome, Microbiota, Metagenomics, 16S rRNA

## Abstract

**Background:**

The study of microbial communities has been revolutionised in recent years by the widespread adoption of culture independent analytical techniques such as 16S rRNA gene sequencing and metagenomics. One potential confounder of these sequence-based approaches is the presence of contamination in DNA extraction kits and other laboratory reagents.

**Results:**

In this study we demonstrate that contaminating DNA is ubiquitous in commonly used DNA extraction kits and other laboratory reagents, varies greatly in composition between different kits and kit batches, and that this contamination critically impacts results obtained from samples containing a low microbial biomass. Contamination impacts both PCR-based 16S rRNA gene surveys and shotgun metagenomics. We provide an extensive list of potential contaminating genera, and guidelines on how to mitigate the effects of contamination.

**Conclusions:**

These results suggest that caution should be advised when applying sequence-based techniques to the study of microbiota present in low biomass environments. Concurrent sequencing of negative control samples is strongly advised.

**Electronic supplementary material:**

The online version of this article (doi:10.1186/s12915-014-0087-z) contains supplementary material, which is available to authorized users.

## Background

Culture-independent studies of microbial communities are revolutionising our understanding of microbiology and revealing exquisite interactions between microbes, animals and plants. Two widely used techniques are deep sequence surveying of PCR-amplified marker genes such as 16S rRNA, or whole-genome shotgun metagenomics, where the entire complement of community DNA is sequenced *en masse*. While both of these approaches are powerful, they have important technical caveats and limitations, which may distort taxonomic distributions and frequencies observed in the sequence dataset. Such limitations, which have been well reported in the literature, include choices relating to sample collection, sample storage and preservation, DNA extraction, amplifying primers, sequencing technology, read length and depth and bioinformatics analysis techniques [1,2].

A related additional problem is the introduction of contaminating microbial DNA during sample preparation. Possible sources of DNA contamination include molecular biology grade water [3–9], PCR reagents [10–15] and DNA extraction kits themselves [16]. Contaminating sequences matching water- and soil-associated bacterial genera including *Acinetobacter, Alcaligenes, Bacillus, Bradyrhizobium, Herbaspirillum, Legionella, Leifsonia, Mesorhizobium, Methylobacterium, Microbacterium, Novosphingobium, Pseudomonas, Ralstonia, Sphingomonas, Stenotrophomonas* and *Xanthomonas* have been reported previously [3–15,17,18]. The presence of contaminating DNA is a particular challenge for researchers working with samples containing a low microbial biomass. In these cases, the low amount of starting material may be effectively swamped by the contaminating DNA and generate misleading results.

Although the presence of such contaminating DNA has been reported in the literature, usually associated with PCR-based studies, its possible impact on high-throughput 16S rRNA gene-based profiling and shotgun metagenomics studies has not been reported. In our laboratories we routinely sequence negative controls, consisting of ‘blank’ DNA extractions and subsequent PCR amplifications. Despite adding no sample template at the DNA extraction step, these negative control samples often yield a range of contaminating bacterial species (see Table 1), which are often also visible in the human-derived samples that are processed concomitantly with the same batch of DNA extraction kits. The presence of contaminating sequences is greater in low-biomass samples (such as from blood or the lung) than in high-biomass samples (such as from faeces), suggesting that there is a critical tipping point where contaminating DNA becomes dominant in sequence libraries.

**Table 1:** **List of contaminant genera detected in sequenced negative ‘blank’ controls**

**Phylum**	**List of constituent contaminant genera**
Proteobacteria	Alpha-proteobacteria:
	*Afipia*, *Aquabacterium* ^e^, *Asticcacaulis*, *Aurantimonas*, *Beijerinckia*, *Bosea*, *Bradyrhizobium* ^*d*^, *Brevundimonas* ^c^, *Caulobacter*, *Craurococcus*, *Devosia*, *Hoeflea* ^e^, *Mesorhizobium*, *Methylobacterium* ^c^, *Novosphingobium*, *Ochrobactrum*, *Paracoccus*, *Pedomicrobium*, *Phyllobacterium* ^e^, *Rhizobium* ^c,d^, *Roseomonas*, *Sphingobium*, *Sphingomonas* ^c,d,e^, *Sphingopyxis*
	Beta-proteobacteria:
	*Acidovorax* ^c,e^, *Azoarcus* ^e^, *Azospira*, *Burkholderia* ^d^, *Comamonas* ^c^, *Cupriavidus* ^c^, *Curvibacter*, *Delftia* ^e^, *Duganella* ^a^, *Herbaspirillum* ^a,c^, *Janthinobacterium* ^e^, *Kingella*, *Leptothrix* ^a^, *Limnobacter* ^e^, *Massilia* ^c^, *Methylophilus*, *Methyloversatilis* ^e^, *Oxalobacter*, *Pelomonas*, *Polaromonas* ^e^, *Ralstonia* ^b,c,d,e^, *Schlegelella*, *Sulfuritalea*, *Undibacterium* ^e^, *Variovorax*
	Gamma-proteobacteria:
	*Acinetobacter* ^a,d,c^, *Enhydrobacter*, *Enterobacter*, *Escherichia* ^a,c,d,e^, *Nevskia* ^e^, *Pseudomonas* ^b,d,e^, *Pseudoxanthomonas*, *Psychrobacter*, *Stenotrophomonas* ^a,b,c,d,e^, *Xanthomonas* ^b^
Actinobacteria	*Aeromicrobium*, *Arthrobacter*, *Beutenbergia*, *Brevibacterium*, *Corynebacterium*, *Curtobacterium*, *Dietzia*, *Geodermatophilus*, *Janibacter*, *Kocuria*, *Microbacterium*, *Micrococcus*, *Microlunatus*, *Patulibacter*, *Propionibacterium* ^e^, *Rhodococcus, Tsukamurella*
Firmicutes	*Abiotrophia, Bacillus* ^b^, *Brevibacillus*, *Brochothrix*, *Facklamia*, *Paenibacillus, Streptococcus*
Bacteroidetes	*Chryseobacterium, Dyadobacter, Flavobacterium* ^d^ *, Hydrotalea, Niastella, Olivibacter*, *Pedobacter, Wautersiella*
Deinococcus-Thermus	*Deinococcus*
Acidobacteria	Predominantly unclassified Acidobacteria Gp2 organisms

The listed genera were all detected in sequenced negative controls that were processed alongside human-derived samples in our laboratories (WTSI, ICL and UB) over a period of four years. A variety of DNA extraction and PCR kits were used over this period, although DNA was primarily extracted using the FastDNA SPIN Kit for Soil. Genus names followed by a superscript letter indicate those that have also been independently reported as contaminants previously. ^a^also reported by Tanner *et al*. [12]; ^b^also reported by Grahn *et al*. [14]; ^c^also reported by Barton *et al*. [17]; ^d^also reported by Laurence *et al*. [18]; ^e^also detected as contaminants of multiple displacement amplification kits (information provided by Paul Scott, Wellcome Trust Sanger Institute). ICL, Imperial College London; UB, University of Birmingham; WTSI, Wellcome Trust Sanger Institute.

Many recent publications [19–37] describe important or core microbiota members, often members that are biologically unexpected, which overlap with previously-described contaminant genera. Spurred by this and by the results from negative control samples in our own laboratories when dealing with low-input DNA samples, we investigated the impact of contamination on microbiota studies and explored methods to limit the impact of such contamination. In this study we identify the range of contaminants present in commonly used DNA extraction reagents and demonstrate the significant impact they can have on microbiota studies.

## Results

### 16S rRNA gene sequencing of a pure *Salmonella bongori* culture

To demonstrate the presence of contaminating DNA and its impact on high and low biomass samples, we used 16S rRNA gene sequence profiling of a pure culture of *Salmonella bongori* that had undergone five rounds of serial ten-fold dilutions (equating to a range of approximately 10^8^ cells as input for DNA extraction in the original undiluted sample, to 10^3^ cells in dilution five). *S. bongori* was chosen because we have not observed it as a contaminant in any of our previous studies and it can be differentiated from other *Salmonella* species by 16S rRNA gene sequencing. As a pure culture was used as starting template, regardless of starting biomass, any organisms other than *S. bongori* observed in subsequent DNA sequencing results must therefore be derived from contamination. Aliquots from the dilution series were sent to three institutes (Imperial College London, ICL; University of Birmingham, UB; Wellcome Trust Sanger Institute, WTSI) and processed with different batches of the FastDNA SPIN Kit for Soil (kit FP). 16S rRNA gene amplicons were generated using both 20 and 40 PCR cycles and returned to WTSI for Illumina MiSeq sequencing.

**Figure 1:**
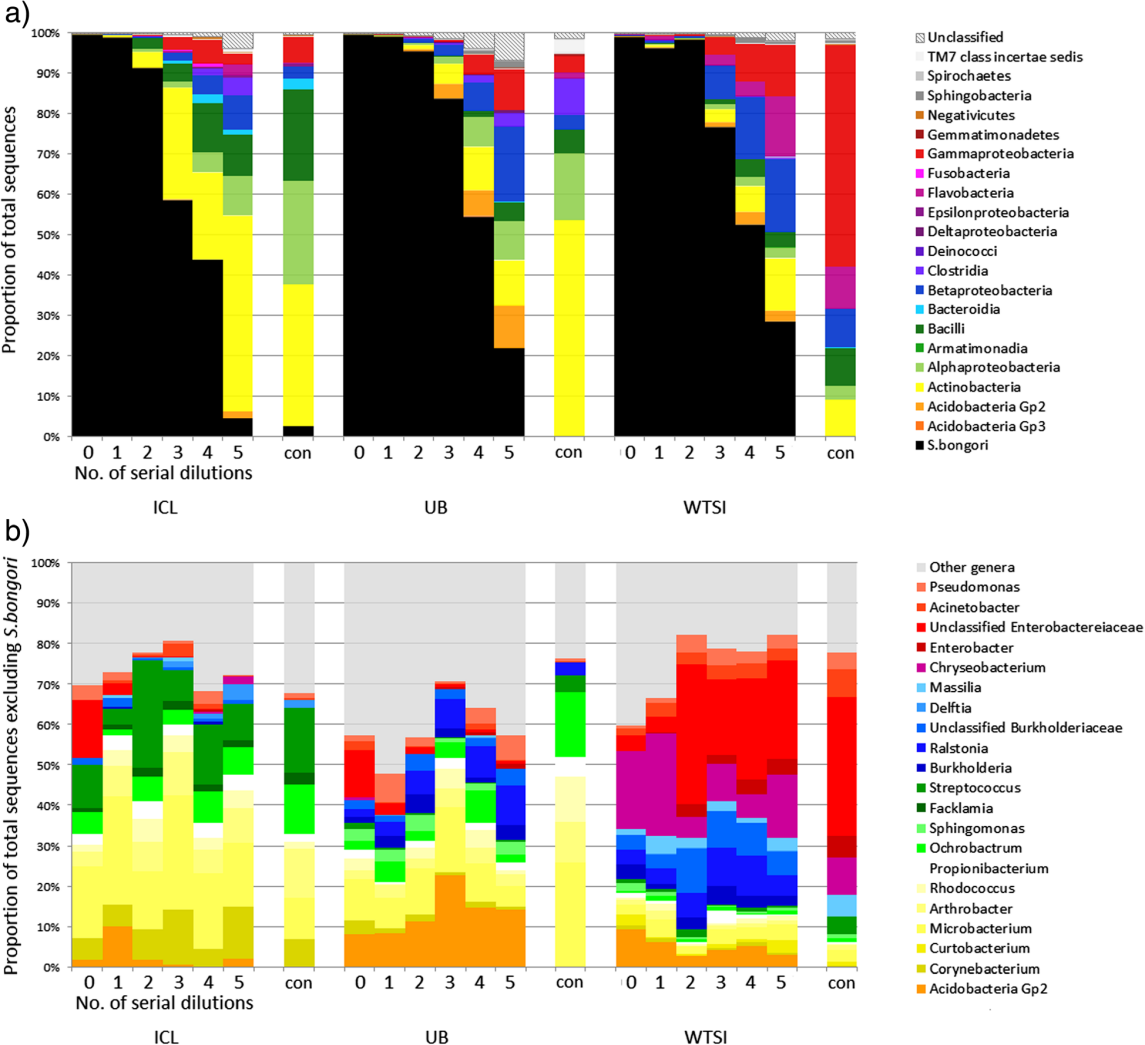
**Summary of 16S rRNA gene sequencing taxonomic assignment from ten-fold diluted pure cultures and controls.** Undiluted DNA extractions contained approximately 10^8^ cells, and controls (annotated in the Figure with 'con') were template-free PCRs. DNA was extracted at ICL, UB and WTSI laboratories and amplified with 40 PCR cycles. Each column represents a single sample; sections **(a)** and **(b)** describe the same samples at different taxonomic levels. **a)** Proportion of *S. bongori* sequence reads in black. The proportional abundance of non-*Salmonella* reads at the Class level is indicated by other colours. As the sample becomes more dilute, the proportion of the sequenced bacterial amplicons from the cultured microorganism decreases and contaminants become more dominant. **b)** Abundance of genera which make up >0.5% of the results from at least one laboratory, excluding *S. bongori*. The profiles of the non-*Salmonella* reads within each laboratory/kit batch are consistent but differ between sites.

*S. bongori* was the sole organism identified in the original undiluted culture but with subsequent dilutions a range of contaminating bacterial groups increased in relative abundance while the proportion of *S. bongori* reads concurrently decreased (Figure 1). By the fifth serial dilution, equivalent to an input biomass of roughly 10^3^*Salmonella* cells, contamination was the dominant feature of the sequencing results. This pattern was consistent across all three sites and was most pronounced with 40 cycles of PCR. These results highlight a key problem with low biomass samples. The most diluted 20-PCR cycle samples resulted in low PCR product yields, leading to under-representation in the multiplexed pool of samples for sequencing as an equimolar mix could not be achieved (read counts for each sample are listed in Additional file 1: Table S1a). Conversely, using 40 PCR cycles generated enough PCR products for effective sequencing (a minimum of at least 14,000 reads per sample were returned, see Additional file 1: Table S1a), but a significant proportion of the resulting sequence data was derived from contaminating, non-*Salmonella*, DNA. It should be noted though that even when using only 20 PCR cycles contamination was still predominant with the lowest input biomass [see Additional file 1: Figure S1].

Sequence profiles revealed some similar taxonomic classifications between all sites, including Acidobacteria Gp2, *Microbacterium, Propionibacterium* and *Pseudomonas* (Figure 1b). Differences between sites were observed, however, with *Chryseobacterium*, *Enterobacter* and *Massilia* more dominant at WTSI, *Sphingomonas* at UB, and *Corynebacterium, Facklamia* and *Streptococcus* at ICL, along with a greater proportion of Actinobacteria in general (Figure 1a). This illustrates that there is variation in contaminant content between laboratories, which may be due to differences between reagent/kit batches or contaminants introduced from the wider laboratory environment. Many of the contaminating operational taxonomic units (OTUs) represent bacterial genera normally found in soil and water, for example *Arthrobacter, Burkholderia, Chryseobacterium, Ochrobactrum, Pseudomonas, Ralstonia, Rhodococcus* and *Sphingomonas*, while others, such as *Corynebacterium, Propionibacterium* and *Streptococcus,* are common human skin-associated organisms. By sequencing PCR ‘blank’ negative controls, specifically PCR-amplified ultrapure water with no template DNA added, we were able to distinguish between taxa that had originated from the DNA extraction kits as opposed to DNA from other sources (such as PCR kit reagents, laboratory consumables or laboratory personnel). Sixty-three taxa were absent from all PCR blank controls but present at >0.1% proportional abundance in one or more serially-diluted *S. bongori* samples [see Additional file 1: Figure S2], suggesting that they were introduced to the samples at the DNA extraction stage. These include several abundant genera observed at all three sites, such as Acidobacteria Gp2, *Burkholderia,* unclassified Burkholderiaceae and *Mesorhizobium*. It also includes taxa, such as *Hydrotalea* and *Bradyrhizobium*, that were only abundant in samples processed by one or two sites, possibly indicative of variation in contaminants between different batches of the same type of DNA extraction kit.

### Quantitative PCR of bacterial biomass

**Figure 2:**
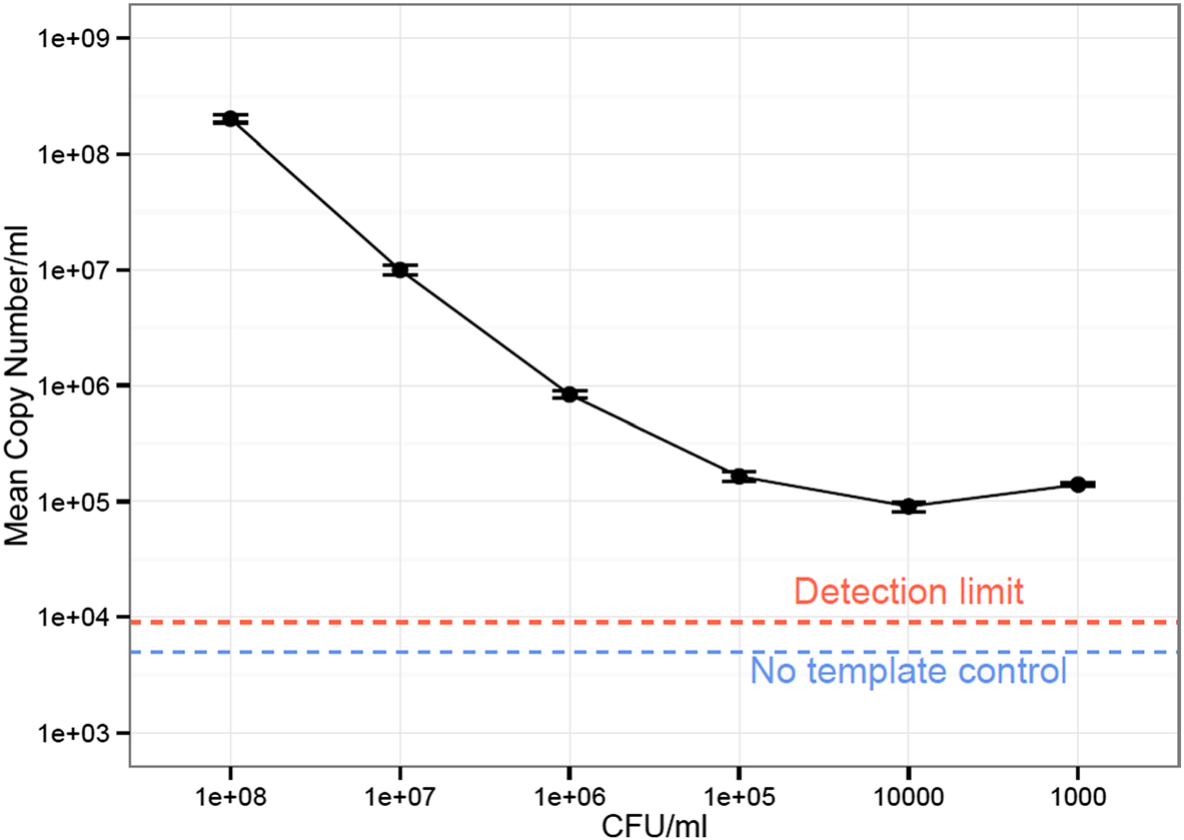
**Copy number of total 16S rRNA genes present in a dilution series of** ***S. bongori*** **culture.** Total bacterial DNA present in serial ten-fold dilutions of a pure *S. bongori* culture was quantified using qPCR. While the copy number initially reduces in tandem with increased dilution, plateauing after four dilutions indicates consistent background levels of contaminating DNA. Error bars indicate standard deviation of triplicate reactions. The broken red line indicates the detection limit of 45 copies of 16S rRNA genes. The no template internal control for the qPCR reactions (shown in blue) was below the cycle threshold selected for interpreting the fluorescence values (that is, less than 0), indicating the contamination did not come from the qPCR reagents themselves.

To assess how much background bacterial DNA was present in the samples, we performed qPCR of bacterial 16S rRNA genes and calculated the copy number of genes present with reference to a standard curve. Assuming a complete absence of contamination, copy number of the 16S rRNA genes present should correlate with dilution of *S. bongori* and reduce in a linear manner. However, at the third dilution copy number remained stable and did not reduce further, indicating the presence of background DNA at approximately 500 copies per μl of elution volume from the DNA extraction kit (Figure 2).

### Shotgun metagenomics of a pure *S. bongori* culture processed with four commercial DNA extraction kits

**Figure 3:**
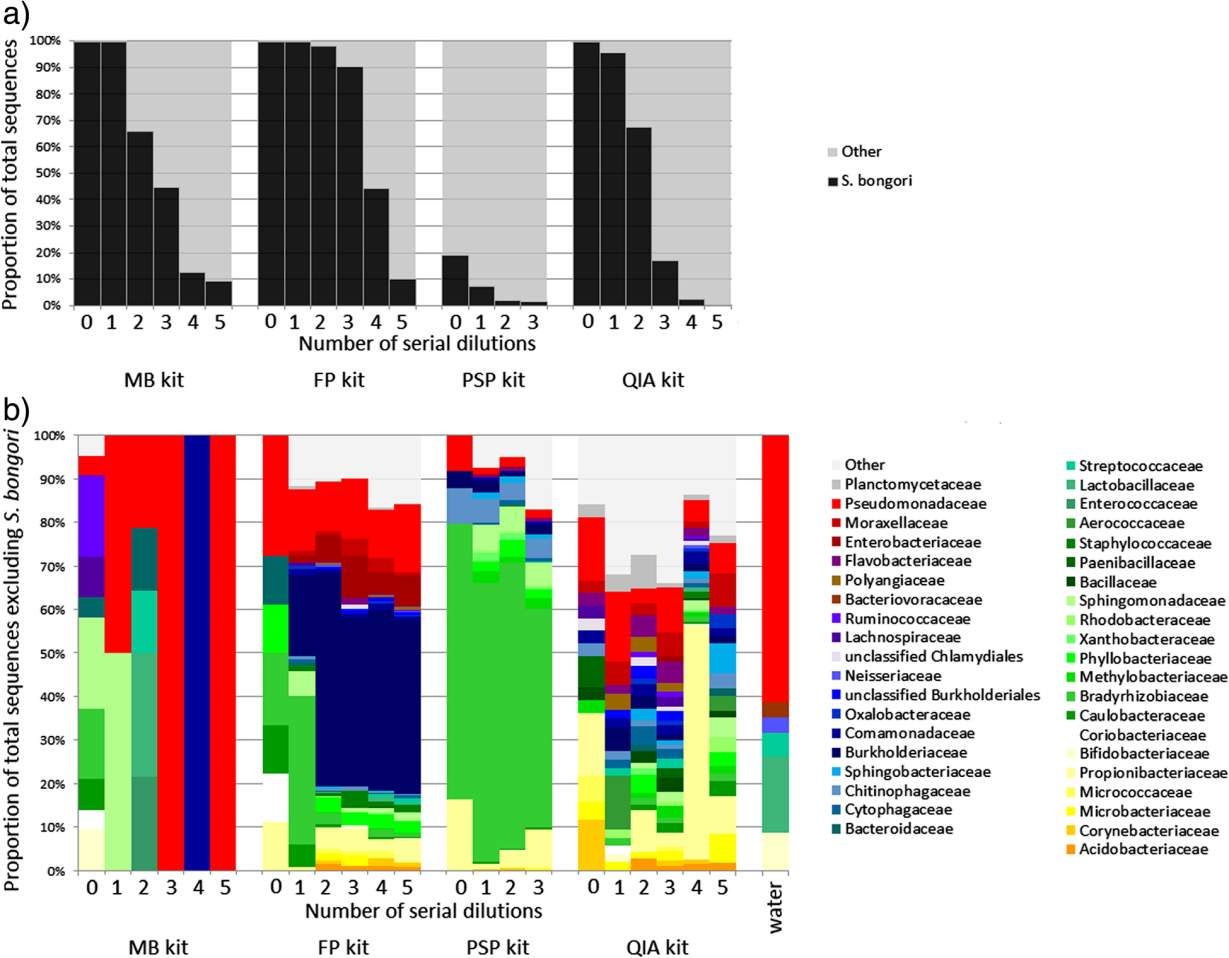
**Summary of the metagenomic data for the** ***S. bongori*** **ten-fold dilution series (initial undiluted samples contained approximately 10** ^**8**^ **cells), extracted with four different kits.** Each column represents a single sample. A sample of ultrapure water, without DNA extraction, was also sequenced (labelled ‘water’). **a)** As the starting material becomes more diluted, the proportion of sequenced reads mapping to the *S. bongori* reference genome decreases for all kits and contamination becomes more prominent. **b)** The profile of the non-*Salmonella* reads (grouped by Family, only those comprising >1% of reads from at least one kit are shown) is different for each of the four kits.

Having established that 16S rRNA gene sequencing results can be confounded by contaminating DNA, we next investigated whether similar patterns emerge in shotgun metagenomics studies, which do not involve a targeted PCR step. We hypothesised that if contamination arises from the DNA extraction kit, it should also be present in metagenomic sequencing results. DNA extraction kits from four different manufacturers were used in order to investigate whether or not the problem was limited to a single manufacturer. Aliquots from the same *S. bongori* dilution series were processed at UB with the FastDNA SPIN Kit for Soil (FP), MoBio UltraClean Microbial DNA Isolation Kit (MB), QIAmp DNA Stool Mini Kit (QIA) and PSP Spin Stool DNA Plus kit (PSP). As with 16S rRNA gene sequencing, it was found that as the sample dilution increased, the proportion of reads mapping to the *S. bongori* reference genome sequence decreased (Figure 3a). Regardless of kit, contamination was always the predominant feature of the sequence data by the fourth serial dilution, which equated to an input of around 10^4^*Salmonella* cells.

Samples were processed concurrently within the same laboratory. If the contamination was derived from the laboratory environment then similar bacterial compositions would be expected in each of the results. Instead, a range of environmental bacteria was observed, which were of a different profile in each kit (Figure 3b). FP had a stable kit profile dominated by *Burkholderia*, PSP was dominated by *Bradyrhizobium*, while the QIA kit had the most complex mix of bacterial DNA. Bradyrhizobiaceae, Burkholderiaceae, Chitinophagaceae, Comomonadaceae, Propionibacteriaceae and Pseudomonadaceae were present in at least three quarters of the dilutions from PSP, FP and QIA kits. However, relative abundances of taxa at the Family level varied according to kit: FP was marked by Burkholderiaceae and Enterobacteriaceae, PSP was marked by Bradyrhizobiaceae and Chitinophagaceae. The contamination in the QIA kit was relatively diverse in comparison to the other kits, and included higher proportions of Aerococcaceae, Bacillaceae, Flavobacteriaceae, Microbacteriaceae, Paenibacillaceae, Planctomycetaceae and Polyangiaceae than the other kits. Kit MB did not have a distinct contaminant profile. This was likely a result of the very low number of reads sequenced, with 210 reads in dilution 2, 79 reads in dilution 3 and fewer than 20 reads in subsequent dilutions [see Additional file 1: Table S1b]. Although read count is only a semi-quantitative measure of DNA concentration, this may indicate that levels of background contamination from this kit were comparatively lower than the other kits tested.

Comparatively few contaminant taxa that were detected in the ‘blank’ water control, which was dominated by *Pseudomonas*, were detected in the serially diluted metagenomic samples. This provided further evidence that the observed contamination was likely to have originated in large part from the DNA extraction kits themselves. These metagenomic results, therefore, clearly show that contamination becomes the dominant feature of sequence data from low biomass samples, and that the kit used to extract DNA can have an impact on the observed bacterial diversity, even in the absence of a PCR amplification step. Reducing input biomass again increases the impact of these contaminants upon the observed microbiota.

### Impact of contaminated extraction kits on a study of low-biomass microbiota

Having established that the contamination in different lots of DNA extraction kits is not constant or predictable, we next show the impact that this can have on real datasets. A recent study in a refugee camp on the border between Thailand and Burma used an existing nasopharyngeal swab archive [38] to examine the development of the infant nasopharyngeal microbiota. A cohort of 20 children born in 2007/2008 were sampled every month until two years of age, and the 16S rRNA gene profiles of these swabs were sequenced by 454 pyrosequencing.

**Figure 4:**
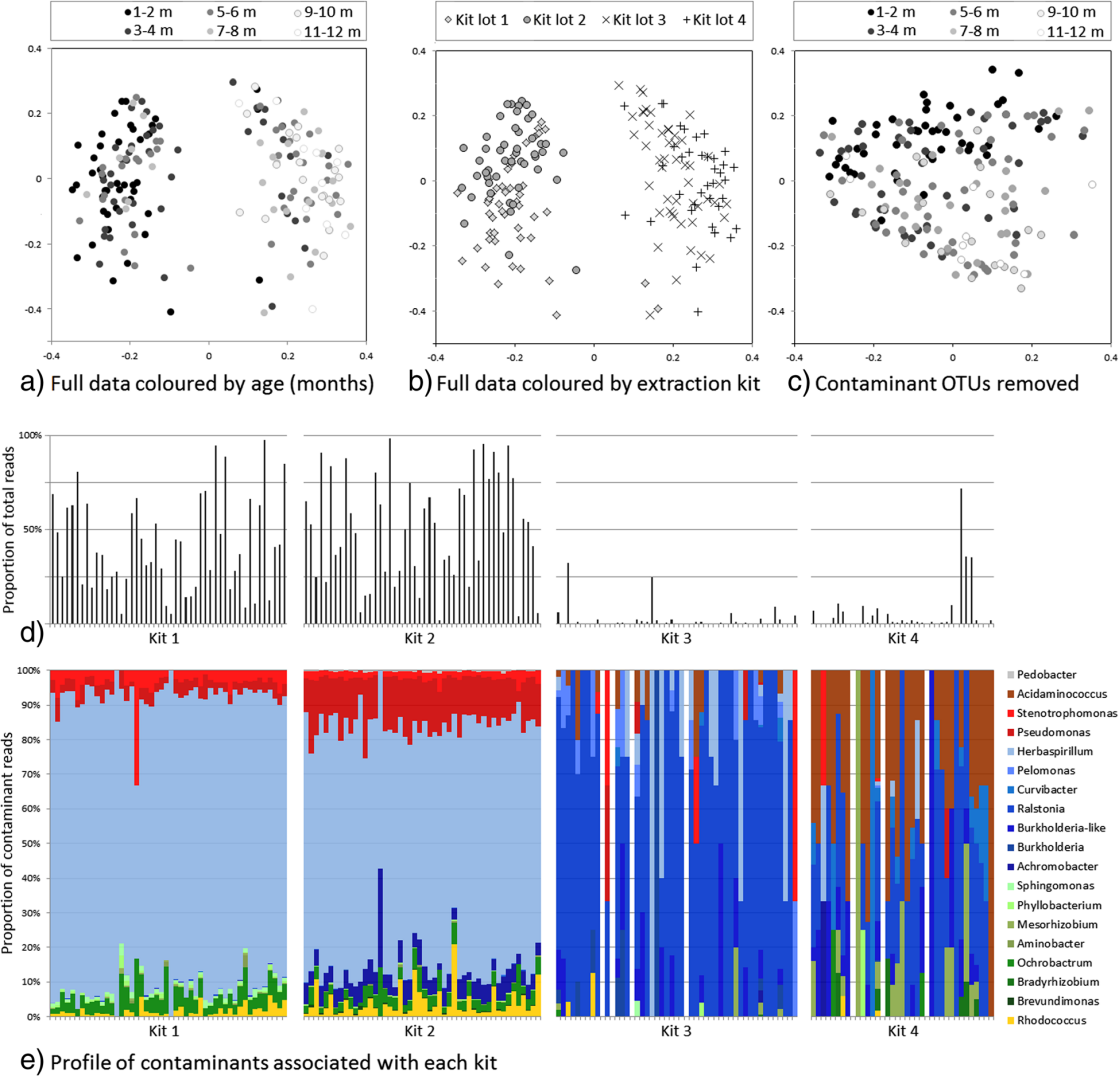
**Summary of the contaminant content of nasopharyngeal samples from Thailand. a)** The PCoA plot appears to show age-related clustering; however, **b)** extraction kit lot explains the pattern better. **c)** When coloured by age, the plot shows the loss of the initial clustering pattern after excluding contaminant OTUs from ordination. **d)** The proportion of reads attributed to contaminant OTUs for each sample, demonstrating that the first two kits were the most heavily contaminated. **e)** Genus-level profile of contaminant OTUs for each kit used.

Principal coordinate analysis (PCoA) showed two distinct clusters distinguishing samples taken during early life from those taken from subsequent sampling time points, suggesting an early, founder nasopharyngeal microbiota (Figure 4a). Four batches of FP kits had been used to extract the samples and a record was made of which kit was used for each sample. Further analysis of the OTUs present indicated that samples possessed different communities depending on which kit had been used for DNA extraction (Figure 4b,d,e) and that the first two kits’ associated OTUs made up the majority of their samples’ reads (Figure 4d). As samples had been extracted in chronological order, rather than random order, this led to the false conclusion that OTUs from the first two kits were associated with age. OTUs driving clustering to the left in Figure 4a and b (*P* value of <0.01), were classified as *Achromobacter, Aminobacter, Brevundimonas, Herbaspirillum*, *Ochrobactrum*, *Pedobacter*, *Pseudomonas*, *Rhodococcus*, *Sphingomonas* and *Stenotrophomonas.* OTUs driving data points to the right (*P* value of <0.01) included *Acidaminococcus* and *Ralstonia*. A full list of significant OTUs is shown in Additional file 1: Table S2. Once the contaminants were identified and removed, the PCoA clustering of samples from the run no longer had a discernible pattern, showing that the contamination was the biggest driver of sample ordination (Figure 4c). New aliquots were obtained from the original sample archive and were reprocessed using a different kit lot and sequenced. The previously observed contaminant OTUs were not detected, further confirming their absence in the original nasopharyngeal samples (manuscript in preparation, Salter S, Turner P, Turner C, Watthanaworawit W, Goldblatt D, Nosten F, Mather A, Parkhill J, Bentley S).

This dataset, therefore, serves as a case study for the significant, and potentially misleading, impact that contaminants originating from kits can have on microbiota analyses and subsequent conclusions.

## Discussion

Results presented here show that contamination with bacterial DNA or cells in DNA extraction kit reagents, and the wider laboratory environment, should not only be a concern for 16S rRNA gene sequencing projects, which require PCR amplification, but also for shotgun metagenomics projects.

Contaminating DNA has been reported from PCR reagents, kits and water many times [3–15,17]. The taxa identified are mostly soil- or water-dwelling bacteria and are frequently associated with nitrogen fixation. One explanation for this may be that nitrogen is often used instead of air in ultrapure water storage tanks [3]. Contamination of DNA extraction kit reagents has also been reported [16] and kit contamination is a particular challenge for low biomass studies, which may provide little template DNA to compete with that in the reagents for amplification [12,39]. Issues of contamination have plagued studies, with high-profile examples in the fields of novel virus discovery, such as in the false association of XMRV and chronic fatigue syndrome [40], and the study of ancient DNA of early humans and pathogens [41,42]. The microbial content of ancient ice core samples has also shown to be inconsistent when analysed by different laboratories [39].

The importance of this issue when analysing low biomass samples, despite such high-profile reports of reagent contamination, apparently remains underappreciated in the microbiota research community. Well-controlled studies, such as in Segal *et al*. who examined the lung microbiota through bronchoalveolar lavage sampling, report their results against the backdrop of copious sequenced ‘background’ controls [43]. However, many recent DNA sequence-based publications that describe the microbial communities of low-biomass environments do not report DNA quantification on initial samples, sequencing of negative controls or describe their contaminant removal or identification procedures. Our literature searches have indicated that there are a number of low biomass microbiota studies that report taxa, often statistically noteworthy or core members, that overlap with those we report here from our negative control kit reagents and water (shown in Table 1). While it is possible that the suspect taxa are genuinely present in these samples, in many cases they are biologically unexpected: for example, rhizosphere-associated bacteria that have been implicated in human disease [27,44]. Tellingly, Laurence *et al.* [18] recently demonstrated with an *in silico* analysis that *Bradyrhizobium* is a common contaminant of sequencing datasets including the 1000 Human Genome Project. Having demonstrated the critical impact that contaminating DNA may have on conclusions drawn from sequence-based data, it becomes important to be able to determine which observations are genuine. For environmental samples, such as soil or water, the problem of identifying contaminants requires special attention as the contaminants may be taxa that are indistinguishable from those genuinely present in the samples.

A number of methods have been devised to treat reagents in order to reduce potential contamination, including: gamma [45] or UV radiation [13,46–48], DNase treatment [10,13,47,49–51], restriction digests [10,13,47,52,53], caesium chloride density gradient centrifugation [10] and DNA intercalation and crosslinking with 8-methoxypsoralen [47,54], propidium monoazide [55] or ethidium monoazide [56,57]. However, tests of these methods show varying levels of success. Radiation may reduce the activity of enzymes, DNase inactivation can also damage the polymerase, restriction enzymes may introduce more contaminating DNA, and unbound DNA intercalators inhibit amplification of the intended template [56,58]. An alternative to decontamination is to preferentially amplify the template DNA using broad range primer extension PCR [59] but this, and the treatment of the PCR reagents, cannot account for contamination introduced through DNA extraction kits.

A simple *in silico* approach for microbiota studies is to identify contaminants that are sequenced using negative controls or contaminant databases in order to screen them out of downstream analysis [17,60]. In the event that contaminating organisms are discovered that are also biologically plausible and should not be excluded from the analysis, alternative approaches could be employed [61]. Statistical approaches and basic visualization to compare relative abundances or rank-order information between negative controls and samples may help determine if taxa are also real. Alternative bioinformatics approaches, such as oligotyping [62], could potentially provide fine-grained discrimination between contaminant OTUs and genuine OTUs assigned to the same genus or species. For shotgun metagenomics studies, use of strain-specific genes or use of phylogenetic information across multiple marker genes may also provide necessary discrimination. Deviation from a neutral model of community formation to compare source (kit controls) and recipient communities may also be useful in this context [63].

By adding negative sequencing controls (specifically, template-free ‘blanks’ processed with the same DNA extraction and PCR amplification kits as the real samples, sequenced on the same run) it is possible to identify reads originating from contamination, and distinguish them from those derived from actual constituent taxa. We have developed a set of recommendations that may help to limit the impact of reagent contamination (Box 1). With awareness of common contaminating species, careful collection of controls to cover different batches of sampling, extraction and PCR kits, and sequencing to monitor the content of these controls, it should be possible to effectively mitigate the impact of contaminants in microbiota studies.

## Conclusions

We have shown that bacterial DNA contamination in extraction kits and laboratory reagents can significantly influence the results of microbiota studies, particularly when investigating samples containing a low microbial biomass. Such contamination is a concern for both 16S rRNA gene sequencing projects, which require targeted PCR amplification and enrichment, and also for shotgun metagenomic projects which do not. Awareness of this issue by the microbiota research community is important to ensure that studies are adequately controlled and erroneous conclusions are not drawn from culture-independent investigations.

## Methods

### Samples

For the 16S rRNA gene and metagenomic profiling, *Salmonella bongori* strain NCTC-12419 was cultured overnight on Luria-Bertani (LB) plates without antibiotics at 37°C. A single colony was used to inoculate an LB broth, which was incubated with shaking at 37°C overnight. The OD_600_ upon retrieval was 1.62, equating to around 10^9^ colony forming units (CFU)/ml. A total of 20 μl from the culture was plated out on LB and observed to be a pure culture after overnight incubation. Five ten-fold dilutions from the starter culture were made in fresh LB. Aliquots (1 ml) of each dilution were immediately stored at −80°C, and duplicates shipped on dry ice to Imperial College London and the University of Birmingham.

For the nasopharyngeal microbiota study, the samples were nasopharyngeal swabs collected from a cohort of infants in the Maela refugee camp in Thailand as described previously [38]. These were vortexed in skimmed milk, tryptone, glucose and glycerin media (STGG) medium and then stored at −80°C.

### DNA extraction

For the 16S rRNA gene profiling work, each of the three institutes (Imperial College London, ICL; University of Birmingham, UB; Wellcome Trust Sanger Institute, WTSI) extracted DNA from the *S. bongori* aliquots in parallel, using different production batches of the FastDNA Spin Kit For Soil (MP Biomedicals, Santa Ana, California, USA kit lots #38098, #15447 and #30252), according to the manufacturer’s protocol. Each aliquot was extracted once at each institute. UB and WTSI extracted DNA from 200 μl of sample and eluted in 50 μl; ICL extracted from 500 μl of sample and eluted in 100 μl. This meant that our DNA extractions across the five-fold serial dilutions spanned a range of sample biomass from approximately 10^8^ down to 10^3^ cells.

For the metagenomic sequencing, 200 μl aliquots of each *S. bongori* dilution were processed using four commercially available DNA extraction kits at UB. The final elution volume for all kits was 100 μl per sample. The FP kit (lot #38098) was used according to the manufacturer’s protocol, with the exception of the homogeniser step. This was performed with a Qiagen Tissue Lyser: one minute at speed 30/second followed by 30 seconds cooling the tubes on ice, repeated three times. The UltraClean Microbial DNA Isolation Kit (MO BIO Laboratories, Carlsbad, California, USA) (kit MB, lot #U13F22) was used according to the manufacturer’s protocol with the exception of homogenisation, which was replaced by 10 minutes of vortexing. The QIAmp DNA Stool Mini Kit (Qiagen, Venlo, Limburg, Netherlands) (kit QIA, lot #145036714) was used according to the manufacturer’s stool pathogen detection protocol. The heating step was at 90°C. The PSP Spin Stool DNA Plus kit (STRATEC Molecular, Birkenfeld, Germany) (kit PSP, lot #JB110047) was used according to the manufacturer’s stool homogenate protocol. Each aliquot was processed once with each kit. All extraction reagents were included with all kits, except for ethanol added for wash steps. In addition to these samples, a negative control was included consisting of ultrapure water that had not been processed with any DNA extraction kit.

For the nasopharyngeal microbiota study, a 200 μl aliquot was taken from each sample and processed with the manufacturer’s vortex modification of the FP kit protocol. DNA was then shipped to WTSI for further processing and sequencing (see below).

### qPCR

A standard curve was produced by cloning the near full-length 16S rRNA gene of *Vibrio natriegens* DSMZ 759 amplified using primers 27 F and 1492R [64] into the TOPO TA vector (Life Technologies, Carlsbad, California, USA), quantifying using fluorescent assay (Quant-IT, Life Technologies) and diluting to produce a standard curve from 10^8^ to 10^3^ copies per μl. A ViiA 7 Real-time PCR system (Life Technologies) with SYBR Fast qPCR Master Mix (KAPA Biosystems, Wilmington, Massachusetts, USA) was used to perform quantitative PCR of the V4 region of the bacterial 16S rRNA gene for each *S. bongori* dilution extraction (which were carried out using the FastDNA SPIN Kit for Soil (MP Biomedicals), kit lot #15447). Primers used were: S-D-Bact-0564-a-S- 15, 5′-AYTGGGYDTAAAGNG and S-D-Bact-0785-b-A-18, 5-TACNVGGGTATCTAATCC [65] generating a 253 bp amplicon. Reactions (15 μl) were performed in triplicate and included template-free controls. Reactions consisted of 0.3 μl of 10 μM dilutions of each primer, 7.5 μl of SYBR Fast mastermix and 1.9 μl of microbial DNA free PCR water (MOBIO) and 5 μl of 1:5 diluted template (to avoid pipetting less than 5 μl). Cycle conditions were 90°C for 3 minutes followed by 40 cycles of: 95°C for 20 seconds, 50°C for 30 seconds, and 72°C for 30 seconds. Melt curves were run from 60 to 95°C over 15 minutes.

### Sequencing

Samples for the *S. bongori* culture 16S rRNA gene profiling were PCR-amplified using barcoded fusion primers targeting the V1-V2 region of the gene (27f_Miseq: AATGATACGGCGACCACCGAGATCTACAC TATGGTAATT CC AGMGTTYGATYMTGGCTCAG and 338R_MiSeq: CAAGCAGAAGACGGCATACGAGAT nnnnnnnnnnnn AGTCAGTCAG AA GCTGCCTCCCGTAGGAGT, where the n string represents unique 12-mer barcodes used for each sample studied, and then sequenced on the Illumina MiSeq platform using 2 × 250 bp cycles. The PCR amplification was carried out with the Q5 High-Fidelity PCR kit (New England Biolabs, Ipswich, Massachusetts, USA) at WTSI, ICL and UB, using fresh reagents and consumables, autoclaved microcentrifuge tubes, filtered pipette tips, and performed in a hood to reduce the risk of airborne contamination. Each sample was amplified with both 20 and 40 PCR cycles under the following conditions: 94°C for 30 seconds, 53°C for 30 seconds, 68°C for 2 minutes. Negative controls in the form of a PCR-amplified ultrapure water sample were included for each batch. PCR products were visualised on an agarose gel: bands were visible for all 40 cycle samples and the first four dilutions of the 20 cycle samples. Data are deposited under ENA project accession EMBL: ERP006737; sample details and individual accession numbers are detailed in Additional file 1: Table S1a.

For metagenomic sequencing, all samples were quantified using Nanodrop (Thermo Scientific, Waltham, Massachusetts, USA) and Qubit (Life Technologies) machines, and did not need to be diluted before Illumina Nextera XT library preparation (processed according to the manufacturer’s protocol). Libraries were multiplexed on the Illumina MiSeq in paired 250-base mode following a standard MiSeq wash protocol. Data are deposited under ENA project accession EMBL: ERP006808. Sample details and individual accession numbers are provided in Additional file 1: Table S1b.

For the nasopharyngeal microbiota study, DNA extractions from 182 swabs were PCR-amplified and barcoded for sequencing the 16S rRNA gene V3-V5 region on the 454 platform as described previously [66].

### Sequence analysis

For the 16S rRNA gene profiling, data were processed using mothur [67]. The mothur MiSeq SOP [68] was followed with the exception of screen.seqs, which used the maximum length of the 97.5 percentile value, and chimera checking, which was performed with Perseus [69] instead of UCHIME. Read counts post-processing and the number of genus-level phylotypes present in each sample are shown in Additional file 1: Table S1a.

For the metagenomic profiling, reads were quality checked and trimmed for low-quality regions and adaptor sequences using Trimmomatic [70]. Similarity sequencing for taxonomic assignments was performed using LAST in six-frame translation mode against the Microbial RefSeq protein database [71]. Taxonomic assignments were determined with MEGAN, which employs a lowest common ancestor (LCA) to taxonomic assignments, using settings Min Support 2, Min Score 250, Max Expected 0.1, Top Percent 10.0 [72].

For the nasopharyngeal microbiota study, the data were processed, cleaned and analysed using the mothur Schloss SOP [73] and randomly subsampled to 200 sequence reads per sample. As part of the contamination identification procedure, the metastats package [74] within mothur was used to identify OTUs that were significantly associated with each extraction kit batch. Jaccard PCoA plots were generated with mothur, comparing the dataset with and without these flagged OTUs included.

## **Box 1**

Recommendations to reduce the impact of contaminants in sequence-based, low-biomass microbiota studies:

1. Maximise the starting sample biomass by choice of sample type, filtration, or enrichment if possible. If microbial load is less than approximately 10^3^ to 10^4^ cells it may not be possible to obtain robust results as contamination appears to predominate. Gram staining, fluorescent *in situ* hybridisation (FISH), qPCR or other forms of DNA quantification prior to amplicon generation/sequencing may be useful guides in this respect. However, it must be noted that the detection limit of microscopy-based techniques may impede accurate quantification of bacterial cell numbers at very low levels, and DNA quantification measures may be impacted by contamination introduced at the DNA extraction stage.
2. Minimise risk of contamination at the point of sample collection. PCR and extraction kit reagents may be treated to reduce contaminant DNA.
3. Collect, process and sequence technical controls from each batch of sample collection/storage medium, each extraction kit, and each PCR kit concurrently with the environmental samples of interest.
4. Samples should be processed in random order to avoid creating false patterns and ideally carried out in replicates, which should be processed using different kit/reagent batches.
5. A record should be made of which sample was processed with which kit so that contamination of a particular kit lot number can be traced through to the final dataset.
6. Quantification of the negative controls and samples should be ongoing during processing in order to monitor contamination as it arises.
7. After sequencing, be wary of taxa that are present in the negative controls, taxa that are statistically associated with a particular batch of reagents, and taxa that are unexpected biologically and also coincide with previously reported contaminants, such as those listed in Table 1.
8. In the event that suspect taxa are still of interest, repeat sequencing should be carried out on additional samples using separate batches of DNA extraction kits/reagents, and, ideally, a non-sequencing-based approach (such as traditional culturing or FISH, using properly validated probe sets) should also be used to further confirm their presence in the samples.

## Additional file

Additional file 1:
**Figure S1 (16S rRNA gene profile of S. bongori amplified with 20 PCR cycles), Figure S2 (genus-level phylotypes that are likely to have originated during DNA extractions), Tables S1a and S1b (accession numbers and read counts for 16S and metagenomic data respectively) and Table S2 (OTUs with significant correlation in Figures** 4 **b and** 4 **c).**
