# Supplementary material for: Reagent and laboratory contamination can critically impact sequence-based microbiome analyses
Source: BMC Biol. 2014 Nov 12;12:87. doi: 10.1186/s12915-014-0087-z (PMC4228153; doi:10.1186/s12915-014-0087-z)
Supplement: Additional file 1: — Figure S1 (16S rRNA gene profile of S. bongori amplified with 20 PCR cycles), Figure S2 (genus-level phylotypes that are likely to have originated during DNA extractions), Tables S1a and S1b (accession numbers and read counts for 16S and metagenomic data respectively) and Table S2 (OTUs with significant correlation in Figures 4 b and 4 c). [file 12915_2014_87_MOESM1_ESM.docx]

**Supplementary Figure S1:**

**16S rRNA gene profile of *S. bongori* pure culture serial dilutions amplified with 20 PCR cycles.**

*S. bongori* is shown in black; other taxa are grouped by Class. Stars mark samples that had fewer than 50 sequence reads: the most diluted samples gave no visible bands on electrophoresis gels after 20 PCR cycles, and these samples therefore tended to be under-represented in the sequence libraries. The centres that performed the DNA extraction and PCR steps are shown at the bottom of the figure (ICL = Imperial College London, UB = University of Birmingham, WTSI = Wellcome Trust Sanger Institute).


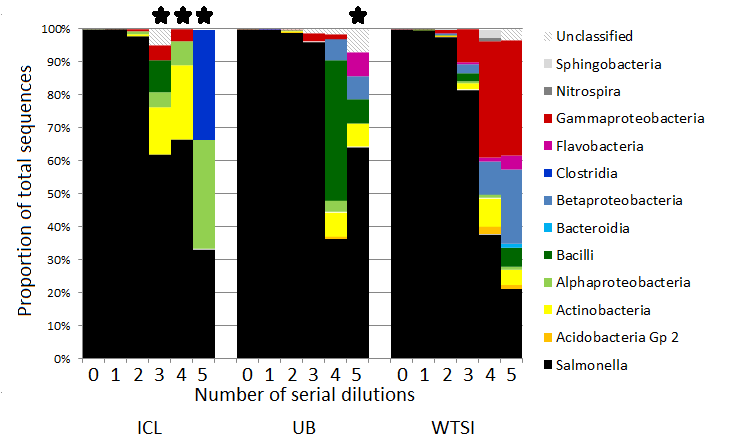


**Supplementary Figure S2:**

Genus-level phylotypes that were absent from all negative controls (ultrapure water not passed through the DNA extraction process) but present at >0.01% proportional abundance in at least one sample. These taxa are unlikely to have been introduced by PCR kit reagents or contamination on the bench, and so are likely to have originated during the DNA extraction process.


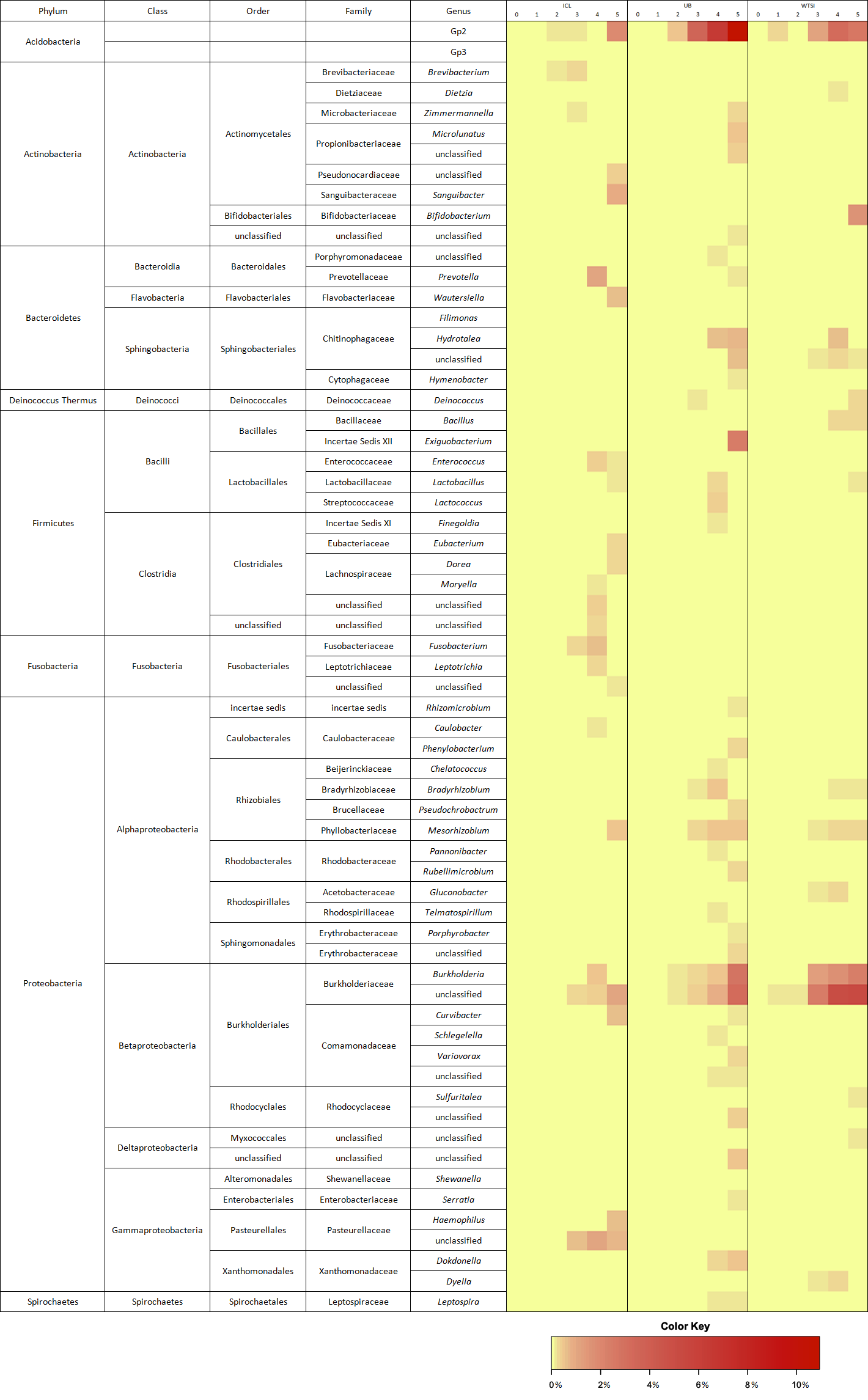
 **Supplementary Table S1a:**

Summary of 16S rRNA gene sequence samples from project accession ERP006737. ICL = Imperial College London, UB = University of Birmingham, WTSI = Wellcome Trust Sanger Institute.

| Sample accession | Run accession | Sample name | Dilution number | Processing Institute | PCR cycles | Post-processing read count | Number of genus-level phylotypes |
| --- | --- | --- | --- | --- | --- | --- | --- |
| ERS532155 | ERR584307 | COX-NF1-20 | 0 (original culture) | ICL | 20 | 33806 | 11 |
| ERS532156 | ERR584308 | COX-NF2-20 | 1 | ICL | 20 | 13210 | 5 |
| ERS532157 | ERR584309 | COX-NF3-20 | 2 | ICL | 20 | 136 | 4 |
| ERS532158 | ERR584310 | COX-NF4-20 | 3 | ICL | 20 | 21 | 9 |
| ERS532159 | ERR584311 | COX-NF5-20 | 4 | ICL | 20 | 27 | 7 |
| ERS532160 | ERR584312 | COX-NF6-20 | 5 | ICL | 20 | 3 | 3 |
| ERS532161 | ERR584313 | COX-NF-NEG-20 | Negative control | ICL | 20 | 6 | 4 |
| ERS532162 | ERR584314 | COX-NF1-40 | 0 | ICL | 40 | 37097 | 36 |
| ERS532163 | ERR584315 | COX-NF2-40 | 1 | ICL | 40 | 47915 | 52 |
| ERS532164 | ERR584316 | COX-NF3-40 | 2 | ICL | 40 | 38116 | 60 |
| ERS532165 | ERR584317 | COX-NF4-40 | 3 | ICL | 40 | 19315 | 53 |
| ERS532166 | ERR584318 | COX-NF5-40 | 4 | ICL | 40 | 14741 | 64 |
| ERS532167 | ERR584319 | COX-NF6-40 | 5 | ICL | 40 | 28856 | 65 |
| ERS532168 | ERR584320 | COX-NF-NEG-40 | Negative control | ICL | 40 | 749 | 29 |
| ERS532169 | ERR584321 | LOM-1-20 | 0 | UB | 20 | 24022 | 7 |
| ERS532170 | ERR584322 | LOM-2-20 | 1 | UB | 20 | 28072 | 21 |
| ERS532171 | ERR584323 | LOM-3-20 | 2 | UB | 20 | 1099 | 9 |
| ERS532172 | ERR584324 | LOM-4-20 | 3 | UB | 20 | 79 | 4 |
| ERS532173 | ERR584325 | LOM-5-20 | 4 | UB | 20 | 137 | 22 |
| ERS532174 | ERR584326 | LOM-6-20 | 5 | UB | 20 | 14 | 6 |
| ERS532182 | ERR584334 | LOM-PCR-20 | Negative control | UB | 20 | 5 | 5 |
| ERS532175 | ERR584327 | LOM-1-40 | 0 | UB | 40 | 57518 | 49 |
| ERS532176 | ERR584328 | LOM-2-40 | 1 | UB | 40 | 38110 | 55 |
| ERS532177 | ERR584329 | LOM-3-40 | 2 | UB | 40 | 49550 | 78 |
| ERS532178 | ERR584330 | LOM-4-40 | 3 | UB | 40 | 49548 | 79 |
| ERS532179 | ERR584331 | LOM-5-40 | 4 | UB | 40 | 43561 | 95 |
| ERS532180 | ERR584332 | LOM-6-40 | 5 | UB | 40 | 24681 | 96 |
| ERS532181 | ERR584333 | LOM-PCR-40 | Negative control | UB | 40 | 44872 | 39 |
| ERS532183 | ERR584335 | SAL-1-20 | 0 | WTSI | 20 | 37322 | 8 |
| ERS532184 | ERR584336 | SAL-2-20 | 1 | WTSI | 20 | 15988 | 17 |
| ERS532185 | ERR584337 | SAL-3-20 | 2 | WTSI | 20 | 2533 | 24 |
| ERS532186 | ERR584338 | SAL-4-20 | 3 | WTSI | 20 | 326 | 27 |
| ERS532187 | ERR584339 | SAL-5-20 | 4 | WTSI | 20 | 82 | 22 |
| ERS532188 | ERR584340 | SAL-6-20 | 5 | WTSI | 20 | 89 | 27 |
| ERS532189 | ERR584341 | SAL-PCR-20 | Negative control | WTSI | 20 | 37 | 15 |
| ERS532190 | ERR584342 | SAL-1-40 | 0 | WTSI | 40 | 44705 | 72 |
| ERS532191 | ERR584343 | SAL-2-40 | 1 | WTSI | 40 | 33092 | 96 |
| ERS532192 | ERR584344 | SAL-3-40 | 2 | WTSI | 40 | 21919 | 60 |
| ERS532193 | ERR584345 | SAL-4-40 | 3 | WTSI | 40 | 29129 | 88 |
| ERS532194 | ERR584346 | SAL-5-40 | 4 | WTSI | 40 | 31304 | 81 |
| ERS532195 | ERR584347 | SAL-6-40 | 5 | WTSI | 40 | 27112 | 112 |
| ERS532196 | ERR584348 | SAL-PCR-40 | Negative control | WTSI | 40 | 29874 | 82 |

**Supplementary Table S1b:**

Accession numbers for WGS metagenomics data (submitted to the ENA under study accession number ERP006808). Samples marked with “CAMBIO” were processed with the MoBio UltraClean Microbial DNA Isolation Kit (termed “MB” in the main text), those marked with “MP_BIO” were processed with the FastDNA SPIN Kit For Soil (termed “FP” in the main text), those marked with “QIAGEN” were processed with the QIAmp DNA Stool Mini Kit (termed “QIA” in the main text), and those marked “PSP_PLUS” were processed with the PSP Spin Stool DNA Plus kit (termed “PSP” in the main text).

| Sample accession | Run accession | Sample Name | Dilution number | Post-processing read count |
| --- | --- | --- | --- | --- |
| ERS534915 | ERR588920 | CAMBIO_1 | 0 (original culture) | 224694 |
| ERS534916 | ERR588921 | CAMBIO_2 | 1 | 98017 |
| ERS534917 | ERR588922 | CAMBIO_3 | 2 | 210 |
| ERS534918 | ERR588923 | CAMBIO_4 | 3 | 79 |
| ERS534919 | ERR588924 | CAMBIO_5 | 4 | 9 |
| ERS534920 | ERR588925 | CAMBIO_6 | 5 | 17 |
| ERS534925 | ERR588930 | MP_BIO_1 | 0 (original culture) | 247020 |
| ERS534926 | ERR588931 | MP_BIO_2 | 1 | 576405 |
| ERS534927 | ERR588932 | MP_BIO_3 | 2 | 541918 |
| ERS534928 | ERR588933 | MP_BIO_4 | 3 | 17674 |
| ERS534929 | ERR588934 | MP_BIO_5 | 4 | 36721 |
| ERS534930 | ERR588935 | MP_BIO_6 | 5 | 7212 |
| ERS534935 | ERR588940 | QIAGEN_1 | 0 (original culture) | 249329 |
| ERS534936 | ERR588941 | QIAGEN_2 | 1 | 93446 |
| ERS534937 | ERR588942 | QIAGEN_3 | 2 | 20553 |
| ERS534938 | ERR588943 | QIAGEN_4 | 3 | 8605 |
| ERS534939 | ERR588944 | QIAGEN_5 | 4 | 12327 |
| ERS534940 | ERR588945 | QIAGEN_6 | 5 | 8109 |
| ERS534945 | ERR588950 | PSP_PLUS_1 | 0 (original culture) | 1548 |
| ERS534946 | ERR588951 | PSP_PLUS_2 | 1 | 12863 |
| ERS534947 | ERR588952 | PSP_PLUS_3 | 2 | 7764 |
| ERS534948 | ERR588953 | PSP_PLUS_4 | 3 | 21415 |
| ERS534949 | ERR588954 | Water | Negative | 310 |

**Supplementary Table S2:**

**OTUs with significant correlation in PCoA plot Figures 4b and 4c.**

All taxa with a p-value of <0.05 are shown, with *P*<0.01 highlighted in bold. Although the data is from human nasopharyngeal swabs, many of the taxa are environmental bacteria associated with the DNA extraction kit.

| OTU identity | Classification | Environmental/human associated | X axis |  | Y axis |  |
| --- | --- | --- | --- | --- | --- | --- |
|  |  |  | Correlation coefficient | p-value | Correlation coefficient | p-value |
| Otu003 | *Herbaspirillum* | Environmental | -0.753 | **0.000** |  |  |
| Otu009 | *Pseudomonas* | Both | -0.591 | **0.000** | 0.250 | **0.001** |
| Otu012 | *Ochrobactrum* | Environmental | -0.568 | **0.000** |  |  |
| Otu014 | *Rhodococcus* | Environmental | -0.486 | **0.000** | 0.198 | **0.007** |
| Otu030 | *Pedobacter* | Environmental | -0.438 | **0.000** | 0.264 | **0.000** |
| Otu040 | *Aminobacter* | Environmental | -0.435 | **0.000** |  |  |
| Otu025 | *Sphingomonas* | Environmental | -0.403 | **0.000** | -0.159 | 0.030 |
| Otu031 | *Brevundimonas* | Environmental | -0.374 | **0.000** | 0.249 | **0.001** |
| Otu015 | *Stenotrophomonas* | Both | -0.366 | **0.000** | -0.163 | 0.027 |
| Otu013 | *Achromobacter* | Environmental | -0.357 | **0.000** |  |  |
| Otu026 | *Phyllobacterium* | Environmental | -0.277 | **0.000** |  |  |
| Otu116 | *Afipia* | Environmental | -0.208 | **0.005** |  |  |
| Otu092 | *Moraxella* | Human | -0.152 | 0.040 |  |  |
| Otu004 | *Haemophilus* | Human | 0.151 | 0.041 |  |  |
| Otu081 | *Pseudonocardia* | Environmental | 0.151 | 0.041 | 0.202 | **0.006** |
| Otu067 | *Bradyrhizobium* | Environmental | 0.154 | 0.037 |  |  |
| Otu007 | *Corynebacterium* | Human | 0.157 | 0.033 |  |  |
| Otu036 | *Burkholderia* | Both | 0.159 | 0.031 |  |  |
| Otu060 | *Curvibacter* | Environmental | 0.164 | 0.026 |  |  |
| Otu016 | *Ralstonia* | Environmental | 0.195 | **0.008** | 0.148 | 0.044 |
| Otu017 | *Acidaminococcus* | Environmental | 0.210 | **0.004** |  |  |
| Otu006 | *Moraxella* | Human | 0.232 | **0.001** |  |  |
| Otu008 | Unclassified *Flavobacteriaceae* | Human | 0.242 | **0.001** |  |  |
| Otu010 | *Helcococcus* | Human | 0.380 | **0.000** |  |  |
| Otu001 | *Moraxella* | Human | 0.404 | **0.000** |  |  |
| Otu197 | *Bordetella* | Both |  |  | 0.147 | 0.046 |
| Otu090 | *Aeromonas* | Environmental |  |  | 0.148 | 0.045 |
| Otu161 | *Kineosphaera* | Environmental |  |  | 0.150 | 0.042 |
| Otu250 | *Perlucidibaca* | Environmental |  |  | 0.153 | 0.038 |
| Otu117 | *Rheinheimera* | Environmental |  |  | 0.153 | 0.038 |
| Otu058 | *Dyella* | Environmental |  |  | 0.153 | 0.038 |
| Otu020 | *Actinobacillus* | Human |  |  | 0.154 | 0.036 |
| Otu251 | Unclassified *Chitinophagaceae* | Environmental |  |  | 0.155 | 0.036 |
| Otu120 | *Veillonella* | Human |  |  | 0.156 | 0.034 |
| Otu113 | *Herbaspirillum* | Environmental |  |  | 0.157 | 0.033 |
| Otu138 | *Perlucidibaca* | Environmental |  |  | 0.157 | 0.033 |
| Otu146 | *Granulicatella* | Human |  |  | 0.161 | 0.029 |
| Otu131 | *Actinomyces* | Both |  |  | 0.162 | 0.028 |
| Otu159 | *Pseudoxanthomonas* | Environmental |  |  | 0.163 | 0.027 |
| Otu094 | *Pseudomonas* | Both |  |  | 0.164 | 0.026 |
| Otu075 | *Wautersiella* | Environmental |  |  | 0.175 | 0.017 |
| Otu115 | *Micrococcus* | Both |  |  | 0.176 | 0.017 |
| Otu072 | *Massilia* | Both |  |  | 0.177 | 0.016 |
| Otu022 | *Acinetobacter* | Both |  |  | 0.178 | 0.016 |
| Otu119 | *Stigmatella* | Environmental |  |  | 0.178 | 0.015 |
| Otu078 | *Paracoccus* | Environmental |  |  | 0.180 | 0.015 |
| Otu091 | *Aeromicrobium* | Environmental |  |  | 0.181 | 0.014 |
| Otu166 | *Arthrobacter* | Environmental |  |  | 0.184 | 0.012 |
| Otu124 | *Moraxella* | Human |  |  | 0.186 | 0.012 |
| Otu055 | *Pseudomonas* | Both |  |  | 0.189 | 0.010 |
| Otu034 | *Janibacter* | Environmental |  |  | 0.189 | **0.010** |
| Otu043 | *Kocuria* | Both |  |  | 0.196 | **0.008** |
| Otu029 | *Brachybacterium* | Human |  |  | 0.202 | **0.006** |
| Otu155 | *Tistrella* | Environmental |  |  | 0.210 | **0.004** |
| Otu054 | *Corynebacterium* | Human |  |  | 0.213 | **0.004** |
| Otu059 | *Luteimonas* | Environmental |  |  | 0.217 | **0.003** |
| Otu095 | *Nocardioides* | Environmental |  |  | 0.227 | **0.002** |
| Otu048 | *Veillonella* | Human |  |  | 0.232 | **0.001** |
| Otu127 | *Nocardioides* | Environmental |  |  | 0.242 | **0.001** |
| Otu046 | *Paracoccus* | Environmental |  |  | 0.244 | **0.001** |
| Otu024 | *Acinetobacter* | Both |  |  | 0.249 | **0.001** |
